# Sustainable and Green Synthesis of Waste-Biomass-Derived Carbon Dots for Parallel and Semi-Quantitative Visual Detection of Cr(VI) and Fe^3+^

**DOI:** 10.3390/molecules27041258

**Published:** 2022-02-13

**Authors:** Lan Xia, Xiuju Li, Yuanhua Zhang, Kai Zhou, Long Yuan, Rui Shi, Kailian Zhang, Qifeng Fu

**Affiliations:** 1 Department of Pharmaceutical Analysis, School of Pharmacy, Southwest Medical University, Luzhou 646000, China; 20200599120020@stu.swmu.edu.cn (L.X.); 20190599120011@stu.swmu.edu.cn (Y.Z.); 20210599120022@stu.swmu.edu.cn (L.Y.); zkl66@swmu.edu.cn (K.Z.); 2 School of Pharmacy, Tongren Polytechnic College, Tongren 554300, China; lxj_trzy@163.com; 3 Analytical and Testing Center, Chongqing University, Chongqing 401331, China; zhoukai2016@cqu.edu.cn; 4 Emergency Department, The Affiliated Traditional Chinese Medicine Hospital of Southwest Medical University, Southwest Medical University, Luzhou 646000, China

**Keywords:** carbon dots, waste biomass, flowers of wintersweet, Cr(VI), Fe^3+^, parallel sensing, visual detection

## Abstract

Carbon dot (CD)-based multi-mode sensing has drawn much attention owing to its wider application range and higher availability compared with single-mode sensing. Herein, a simple and green methodology to construct a CD-based dual-mode fluorescent sensor from the waste biomass of flowers of wintersweet (FW-CDs) for parallel and semi-quantitative visual detection of Cr(VI) and Fe^3+^ was firstly reported. The FW-CD fluorescent probe had a high sensitivity to Cr(VI) and Fe^3+^ with wide ranges of linearity from 0.1 to 60 µM and 0.05 to 100 µM along with low detection limits (LOD) of 0.07 µM and 0.15 µM, respectively. Accordingly, the FW-CD-based dual-mode sensor had an excellent parallel sensing capacity toward Cr(VI) and Fe^3+^ with high selectivity and strong anti-interference capability by co-using dual-functional integration and dual-masking strategies. The developed parallel sensing platform was successfully applied to Cr(VI) and Fe^3+^ quantitative detection in real samples with high precision and good recovery. More importantly, a novel FW-CD-based fluorescent hydrogel sensor was fabricated and first applied in the parallel and semi-quantitative visual detection of Cr(VI) and ferrous ions in industrial effluent and iron supplements, further demonstrating the significant advantage of parallel and visual sensing strategies.

## 1. Introduction

Over the past 10 years, fluorescent carbon dots (CDs) have gained much attention in many areas such as photocatalysis [1,2], chemical sensing [3,4], biological imaging [5,6] and drug delivery [7], which have benefited from their unique advantages, including excellent fluorescence performance, good biocompatibility and easily modifiable functionalities [8,9]. A range of synthesis approaches such as electrochemistry and the chemical oxidation of bulk carbon precursors [10,11], and the hydrothermal [8,12,13], solvothermal [14], ultrasonication [3] and microwave [15] treatment of molecular precursors have been explored for the synthesis of CDs. Among them, hydrothermal synthesis has aroused wider attention due to its benefits of convenient operation, strong practicability and the “green” nature of water [16]. In particular, the conversion of biomass into high-value carbon nanomaterials is conducive to alleviating the energy material crisis and disposing of waste reasonably. The studies on the hydrothermal preparation of “green” CDs from diverse waste biomass precursors (e.g., cotton stalk [17], durian [18], turtle shells [19] and Artemisia argyi leaves [20]) and their various potential applications have drawn considerable attention because of the inherent advantages of biomass over artificial precursors, such as their low cost, good renewability and abundant resources. Besides, since the biomass generally contain abundant carbohydrates, flavones and other natural ingredients, various elements (especially C, O and N) and functional groups could be self-doped and integrated on the as-prepared “green” CDs after hydrothermal treatment. Therefore, multiple recognition and sensing functions might be integrated in one “green” CD-based fluorescent sensor through variant fluorescence-quenching [21] or -enhancement [22] mechanisms, which would endow “green” CDs with a wider application range and higher availability. Moreover, the phenomena of fluorescence-quenching or -enhancement of CDs could be visualized through cost-effective and portable devices such as fluorescent test paper and hydrogel to realize the visual and rapid detection of various analytes, further promoting the application value of “green” CDs.

Up to now, most of the previously reported CD-based sensors (including “green” CD-based sensors) could only detect one kind of analyte such as Fe^3+^ [23], Hg^2+^ [24], Ag^+^ [4], ClO^−^ [25], S^2−^ [26], tetracycline [27] and 4-nitrophenol [28]. By contrast, only a few studies have reported CD-based multifunctional sensors for the multi-mode sensing of two or more types of analytes [29,30,31,32]. For instance, Wang’s group utilized the “green” CDs synthetized by hydrothermal treatment of chitosan and tartaric acid for the continuous fluorescent determination of Fe^3+^ and L-ascorbic acid [33]. However, the majority of the reported CD-based multifunctional fluorescent sensors followed a tandem sensing process [34], which meant that the selective response to the latter analyte could not be achieved without the prerequisite of successful sensing for the former analyte. Consequently, the close, mutual dependence of the sensing mechanisms and effects for multiple analytes would inevitably complicate the analysis procedure and significantly reduce the practicability of CD-based multi-mode detection. Alternatively, the parallel sensing strategy, which means that the selective detection of each analyte is performed independently based on different mechanisms [29,30,32], is a more attractive and efficient option for developing a new generation of CD-based multifunctional sensors. Unfortunately, the research and application of a parallel sensing process for CD-based multifunctional sensors are still at their early stages and the highly independent detection of each analyte without mutual interference remains a great challenge. Therefore, it is essential to explore the huge potential of biomass-derived “green” CDs to fabricate CD-based multifunctional sensors for parallel detection with high specificity.

Wintersweet (*Chimonanthus praecox*), the flowers of which have a strong fragrance, is wildly cultivated as a garden plant and possesses high ornamental and economic value [35]. The abundant natural constituents contained in the waste biomass of flowers of wintersweet (FW), such as sesquiterpenoids, monoterpenes and phenolic compounds [36], may be the ideal precursor sources for hydrothermal treatment, which will endow the synthetic products with multiple elements and functional groups. Therefore, it is reasonable to conjecture that the possible “green” CDs prepared by hydrothermal treatment of FW may simultaneously have multiple recognition and sensing functions for different analytes [37,38,39]. However, to our knowledge, there is no research aimed at exploring the potential application of FW as the novel carbon source for fabricating multifunctional “green” CDs.

The pollution caused by the excessive discharge of heavy metal ions has a strong adverse effect on environmental safety and human health [40]. Among various kinds of toxic metal ions, Cr(VI) has been one of the most common and hazardous pollutants because it is widely found in industrial sewage and is highly toxic for biochemical systems. The toxicity of Cr(VI) is identified to be much higher than that of Cr(III) and the strong solubility of Cr(VI) also increases its transport rate and potential risks [41]. Meanwhile, as an important essential trace element, iron plays a vital role in many biochemical pathways of human beings and other living organisms. The lack or excess of Fe^3+^ in the human body can disturb the body’s balance and metabolism or even trigger many physiological diseases [42]. Although a few analytical techniques for the determination of trace Cr(VI) and Fe^3+^ ions have already been developed, high costs and complex operations have severely hindered their extensive application. Alternatively, a few studies for the determination of Cr(VI) and Fe^3+^ based on fluorescent CDs have been reported [43,44,45]. However, these studies exhibit relatively poor sensitivity for Cr(VI) and Fe^3+^, and cannot effectively address the mutual interference of the analytes when both of them coexist. Besides, none of them have been applied for the visual detection of Cr(VI) and Fe^3+^. Therefore, it is significative to further develop a simple, inexpensive and high-efficiency visual sensing strategy based on fluorescent CDs for the simultaneous detection of Cr(VI) and Fe^3+^ without mutual interference.

**Scheme 1:**
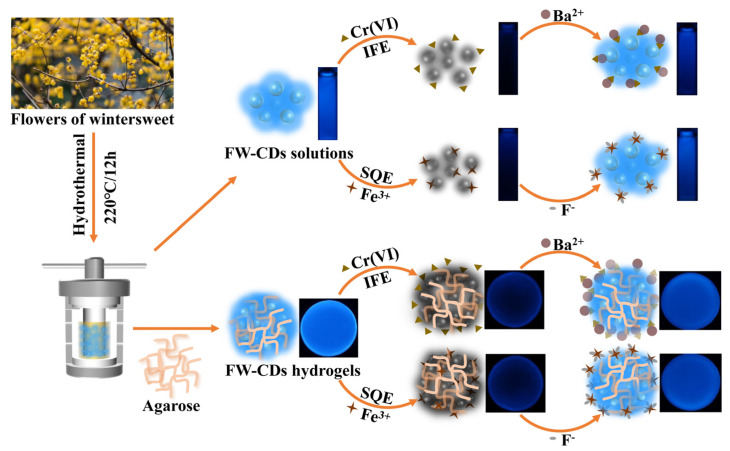
Schematic of the parallel sensing mechanism and application of FW-CD-based fluorescent hydrogel sensor.

Herein, we firstly report a simple and green methodology to construct a novel FW-derived “green” CD (FW-CD)-based multifunctional sensors for the parallel and semi-quantitative visual detection of Cr(VI) and Fe^3+^ with high specificity and sensitivity (Scheme 1). Additionally, the strong availability of FW-CDs for the quantitative measurement of Cr(VI) in industrial sewage and ferrous ion in oral iron supplements was verified. More interestingly, FW-CDs were successfully employed for fabricating a novel FW-CD-based fluorescent hydrogel sensing platform that can realize the parallel and semi-quantitative visual determination of Cr(VI) and ferrous ion in industrial effluent and oral iron supplements, significantly improving its practicability for multi-sensing application. To the best of our knowledge, it is the first time that a parallel visual sensing strategy for the non-interfering, simultaneous and semi-quantitative fluorescent detection of multiple analytes based on one CD-based fluorescent sensor has been reported.

## 2. Materials and Methods

### 2.1. Materials

The flowers of wintersweet were acquired from Southwest Medical University (Luzhou, China) and dried at room temperature. Agarose and quinine sulfate dihydrate were procured from Adamas Reagent Co., Ltd. (Shanghai, China). NaF, BaCl_2_, KCl, NaCl, FeCl_2_·5H_2_O, FeCl_3_·6H_2_O, CuSO_4_·5H_2_O, K_2_Cr_2_O_7_, AgNO_3_ and other salts were procured from Kelong Reagent Co., Ltd. (Sichuan, China). Oral ferrous sulfate tablets (trade name: Nature Made Iron) were purchased from a local drugstore (Luzhou, China). Dialysis membranes (MWCO 1000 Da) were purchased from Yuanye Biotechnology Co., Ltd. (Sichuan, China). The ultrapure water was prepared by an ultrapure water system (Aquaplore, AWL-1002-H).

### 2.2. Characterization

The fluorescence response and UV absorption behavior were observed using an LS55 luminescence spectrometer (Perkin-Elmer, Waltham, MA, USA) and UV–visible spectrophotometer (UV-2600, Shimadzu, Kyoto, Japan). The transmission electron microscopy (TEM) images of FW-CDs were obtained by a JEM-1200EX electron microscope with an accelerating voltage of 120 kV. Besides this, high-resolution TEM (HR-TEM) images were acquired through an FEI Tecnai G2 F20 S-Twin electron microscope. The chemical composition of the FW-CDs was analyzed by an ESCALAB 250Xi X-ray photoelectron spectrometer (XPS, Thermo Electron, Waltham, MA, USA). The Fourier transform infrared (FTIR) spectra were obtained using an FT-IR spectrophotometer (Shimadzu, Japan). The X-ray diffraction (XRD) results were obtained using a Bruker D8 ADVANCE diffractometer. Keeping the excitation wavelength at 375 nm, the fluorescence decay time was monitored using a luminescence spectrometer (FLS1000, Edinburgh, Livingston, UK). An Agilent 730 ICP-OES system was utilized to determine the Cr(VI) and Fe^3+^ concentrations in environmental water samples, iron supplements and industrial effluent.

### 2.3. Synthesis and Purification of Fluorescent FW-CDs

The flowers of wintersweet were utilized as green precursors to synthesize FW-CDs through a convenient one-step hydrothermal treatment. Firstly, the flowers of wintersweet were dried at room temperature before being smashed into a powder. Subsequently, 1 g powder and 30 mL deionized water were added to 100 mL Teflon reactor. After the reaction was completed under the condition of 220 °C for 12 h, the reactor was naturally cooled down to 25 °C and the obtained brown liquid product was dialyzed with a dialysis membrane (MWCO 1000 Da) for over 48 h. The obtained solution after dialysis was further purified through a 0.22 µM filter membrane. Finally, the lyophilized powder was obtained by freeze drying and the as-prepared FW-CDs were kept in a refrigerator (4 °C) for additional characterization and detection.

### 2.4. Fluorescence Detection of Cr(VI) and Fe^3+^

The sensing capability of FW-CDs for Cr(VI) and Fe^3+^ was evaluated by adding a series of different concentrations of Cr(VI) and Fe^3+^ standard solution (0–500 µM) into a quartz cuvette containing 50 µL FW-CDs solution, respectively. The mixture solution was diluted with ultrapure water to a volume of 4 mL. After one minute of the incubation process at room temperature, the fluorescence spectra of the above solutions were recorded under 375 nM. For the selectivity study of FW-CDs to various ions, several other ions (including K^+^, Na^+^, Fe^2+^, Cu^2+^, Ag^+^, Pb^2+^, Cd^2+^, Co^2+^, Ni^2+^, Mn^2+^, Zn^2+^, Mg^2+^, Ba^2+^, Ca^2+^, Al^3+^, Hg^2+^, Cl^−^, CO_3_^2−^, S_2_O_3_^2−^, IO_4_^−^ and SO_4_^2−^) were mixed with the FW-CD solution. All the operations were performed utilizing the same methods as above at room temperature and repeated in triplicate.

### 2.5. Assays of Cr(VI) and Fe^3+^ in Real Samples

Three kinds of environmental water samples, including tap water, lake water and river water, which were, respectively, collected from our laboratory, Yanyu Lake and Tuojiang River (Luzhou, Sichuan, China), were used to evaluate the feasibility of FW-CDs for dual-sensing Cr(VI) and Fe^3+^ in real samples. The standard solutions of Cr(VI) and Fe^3+^ with a series of concentrations were severally added to the real specimens. Before calculating the recovery of the added ions based on the constructed sensing strategy, the residual chlorine and the insoluble impurities in the spiked tap water specimen were eliminated by boiling and filtering through a membrane filter (0.22 µM). Meanwhile, the insoluble impurities in the spiked lake and river water specimens were removed by centrifuging and filtering. The industrial effluent was treated in the same way as above to remove the insoluble impurities. Next, the standard curve obtained from the FW-CD-based sensing system was used to calculate the Cr(VI) concentration in the diluted industrial effluent. As for the determination of Fe^2+^ content in the oral iron supplement, 400 mg of tablet powders were first treated with 20 mL hydrogen peroxide aqueous solution (500 µM, pH = 3) to completely oxidize Fe^2+^ into Fe^3+^. Subsequently, the insoluble impurities were removed through centrifugation and filtration. The resultant supernatant was diluted 100-fold with hydrochloric solution to detect Fe^3+^ based on the developed sensing system.

### 2.6. Preparation of FW-CD-Based Fluorescent Hydrogel Sensor

On the basis of dipole-dipole interactions and hydrogen bonding formed in the reaction process between agarose and the FW-CD solution, the FW-CD-based hydrogel sensor was constructed for the determination of Cr(VI) and Fe^3+^. Briefly, 0.15 g agarose and 10 mL FW-CD solution was well mixed and boiled for 5 min to ensure the complete dissolution of agarose. After that, the well-mixed solution was poured into circular molds to fabricate the FW-CD-doped hydrogel slices (about 1 mg FW-CDs/tablet) with a diameter of about 1.5 cm and a height of about 2 mm. The obtained FW-CD-based fluorescent hydrogel slices were used for the subsequent determination of metal ions in water specimens.

## 3. Results and Discussion

### 3.1. Characterization of FW-CDs

**Figure 1:**
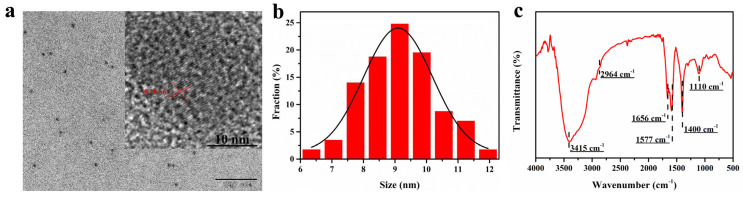
(**a**) TEM and HR-TEM (inset view) images of FW-CDs. (**b**) The size distribution of FW-CDs. (**c**) FTIR spectra of FW-CDs.

The physical structures of the FW-CDs were systematically recorded by the measurements of TEM and XRD. The TEM image (Figure 1a) indicated that the prepared FW-CDs were monodispersed and quasi-spherical with a size distribution ranging from 6 to 12 nm and an average diameter of 9.38 nm (Figure 1b). The clearer image of HR-TEM (Figure 1a inset) showed a lattice spacing at around 0.18 nm in the FW-CDs, which could be attributed to the (102) facet of graphite [46], confirming their graphite-like crystalline structure. The XRD pattern of FW-CDs (Supplementary Figure S1) exhibited a broad diffraction peak at around 20°, corresponding to the graphite-like crystalline peak of the FW-CDs.

The FTIR spectroscopy was operated to measure the functional groups on the surface of the FW-CDs. As depicted in Figure 1c, a strong characteristic peak for the stretching vibration of the O-H or N-H groups [47] and a weak absorption band for the asymmetric stretching vibration of the -CH_2_ group [48] were observed at 3415 and 2964 cm^−1^, respectively. Besides this, the peaks that appeared at 1656 and 1577 cm^−1^ corresponded to the vibration of the C=O [49] and C=C [50] groups in the benzene ring, respectively. Moreover, the other peaks at 1400 and 1110 cm^−1^ indicated the existence of the -COOH and C-O [51] groups, separately. Therefore, the FTIR spectrum indicated that the surface of the FW-CDs possessed abundant and various functional groups, as expected, which laid the foundation for the possible multiple sensing functions of FW-CDs and greatly improved the water solubility and stability of the FW-CDs in aqueous solution.

The elemental composition and functional groups of the FW-CDs were also identified by XPS. Three main peaks at 284.8 eV, 531.1 eV and 399.3 eV were presented by the XPS spectrum (Supplementary Figure S2a) corresponding to C 1s (69.78%), O 1s (24.85%) and N 1s (5.3%), respectively. The deconvolution peaks of C 1s (Supplementary Figure S2b) could be attributed to the structure of the C-O/C-N (285.6 eV) [52,53], C-C/C=C (284.3 eV) [54] and C=O (287.4 eV) bonds [53], respectively. The high-resolution XPS spectrum of O 1s (Supplementary Figure S2c) exhibited three peaks at 530.3 eV, 513.6 eV and 534.8 eV, which were associated with the -OH [55], C=O [3] and C-OH bonds [56], indicating that plenty of hydrophilic groups, such as carboxyl and hydroxyl groups, were integrated into the FW-CDs. In addition, the three deconvolved peaks of N 1s (Supplementary Figure S2d) at 398.38 eV, 400.28 eV and 401.08 eV disclosed that the nitrogen atoms severally existed in the form of pyrrolic-like N, pyridinic-like N and graphitic-like N [57]. In brief, the results of the XPS and FTIR analysis were consistent, confirming again the successful integration of multiple functional groups in the prepared FW-CDs.

### 3.2. Optical Properties and Photostability of FW-CDs

**Figure 2:**
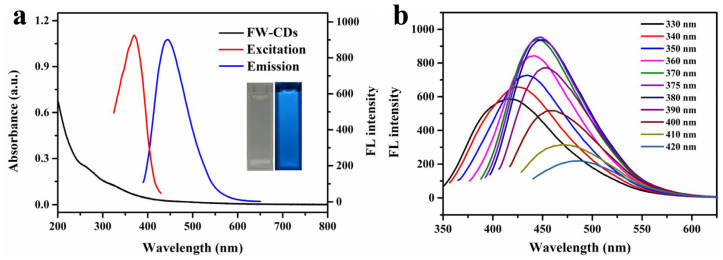
(**a**) UV–vis absorption spectrum (inset at the left: photograph taken under visible light) and optimal excitation/emission fluorescence spectra (inset at the right: photograph taken under 365 nm UV light) of FW-CD aqueous solution. (**b**) Fluorescence spectra of FW-CD aqueous solution upon excitation under different wavelengths.

UV-vis and fluorescence spectra were investigated to further observe the optical behavior of the FW-CDs. The UV–vis spectrum of the FW-CDs (the black line in Figure 2a) showed two absorption peaks centered at 258 nm and 330 nm, which might be caused by the π-π* energy transition of conjugated C=C bonds [58] and the n-π* transition of the structure of the C=O/C=N groups [59]. On the other hand, Figure 2a shows that the FW-CDs had the strongest fluorescent emission at 445 nm under 375 nm excitation. Accordingly, as shown in the inset of Figure 2a, the FW-CDs solution was nearly colorless under visible light, while it exhibited a bright blue fluorescence when illuminated with a UV lamp (365 nm). Additionally, the fluorescence response behavior of the FW-CDs was further explored. The excitation and emission of FW-CDs was measured in the range of 330–420 nm. As exhibited in Figure 2b, an obvious red-shift of the emission wavelength of FW-CDs appeared when the excitation wavelength increased with the interval of 10 nm. This excitation-dependent phenomenon might be assigned to the heterogeneous structure and surface state of the FW-CDs [60]. Additionally, the fluorescence quantum yield of FW-CDs was calculated to be 14.8% using the reference quinine sulfate.

Next, the influences of pH and the concentration of NaCl and UV irradiation on the response intensity of FW-CDs were explored intensively. As illustrated in Supplementary Figure S3, the fluorescence intensity of the FW-CDs was almost consistent under various operation environments, indicating that the FW-CDs had superior photostability in different extreme situations.

### 3.3. Fluorescence Analysis of FW-CDs to Cr(VI) and Fe^3+^

**Figure 3:**
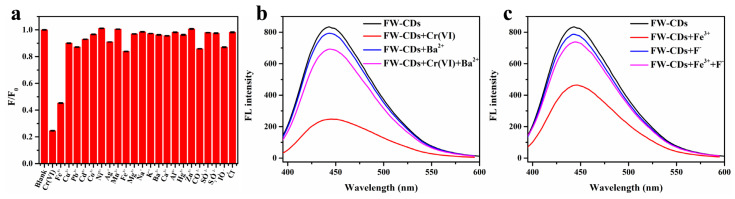
(**a**) Fluorescence intensity ratios (F/F_0_) of FW-CDs before and after the addition of various ions (300 μM). (**b**) Fluorescence intensity changes of FW-CDs in the presence of 400 μM Cr^6+^ and/or 30 mM BaCl_2_. (**c**) Fluorescence intensity changes of FW-CDs in the presence of 400 μM Fe^3+^ and/or 0.1 M NaF. (The error bar was not obvious due to the small fluctuation of data).

Encouraged by a large number of functional groups existing on the surfaces of FW-CDs and the previous works indicating that many waste-biomass-derived CDs had a selective fluorescence response towards metal ions, we speculated that FW-CDs could also be utilized for sensing metal ions. In order to confirm this conjecture, different metal ions and anions of the same concentration (300 µM) were added for exploring the ion selectivity of FW-CDs, including K^+^, Na^+^, Fe^2+^, Fe^3+^, Cu^2+^, Ag^+^, Pb^2+^, Cd^2+^, Co^2+^, Ni^2+^, Mn^2+^, Zn^2+^, Mg^2+^, Ba^2+^, Ca^2+^, Al^3+^, Hg^2+^, Cr(VI), Cl^−^, CO_3_^2−^, S_2_O_3_^2−^, IO_4_^−^ and SO_4_^2^^−^. As depicted in Figure 3a, except for Fe^3+^ and Cr(VI), there was almost no significant fluorescence-quenching caused by other ions, demonstrating that the as-prepared FW-CDs had great potential to be applied in the specific assay of Cr(VI) and Fe^3+^.

Independent analysis of the analytes with high specificity is a challenge because of the similar fluorescence-quenching abilities of Cr(VI) and Fe^3+^. Fortunately, BaCl_2_ and NaF could be used to mask Cr(VI) and Fe^3+^, respectively, in order to achieve independent analysis with high specificity. It can be observed in Figure 3b that the fluorescence intensity of the FW-CD aqueous solution in the presence of 400 μM Cr(VI) could be well recovered after adding an extra 30 mM Ba^2+^ and incubating for 20 min, which could be attributed to the formation of insoluble BaCrO_4_ precipitation. Furthermore, due to the strong chelating effect of F^−^ on Fe^3+^, the fluorescence of FW-CD aqueous solution, which was pre-mixed with 400 μM Fe^3+^, could be commendably recovered after adding 0.1 M F^−^ (Figure 3c). Therefore, the mutual interference between Cr(VI) and Fe^3+^ when they coexisted in the tested specimen could be effectively eliminated via the dual-masking strategy. In the subsequent study, BaCl_2_ and NaF were utilized, respectively, as the specific masking agents to achieve the parallel and independent detection of Cr(VI) and Fe^3+^ with high specificity.

**Figure 4:**
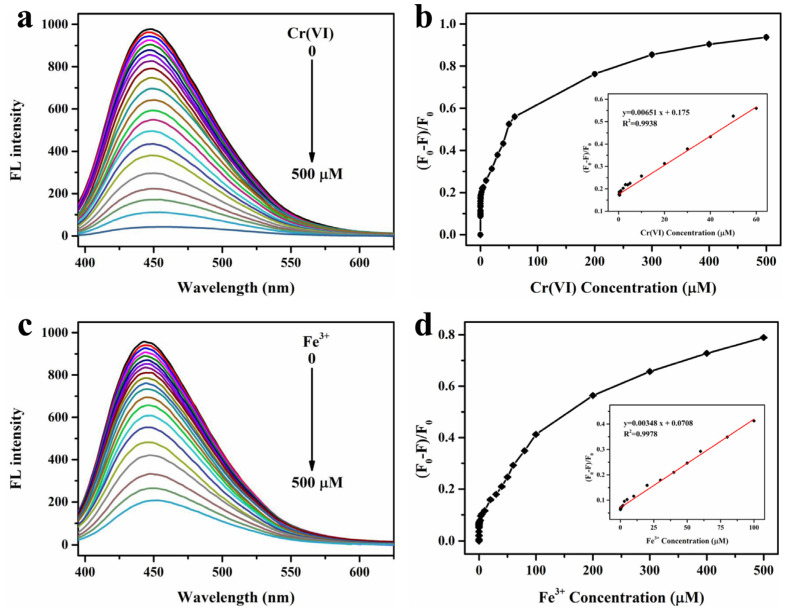
Fluorescence spectra of FW-CDs’ response to Cr(VI) (**a**) and Fe^3+^ (**c**) with different concentrations (from 0 to 500 μM) under excitation at 375 nm; plot for fluorescent quenching ratio ((F_0_ − F)/F_0_) versus Cr(VI) concentration (**b**) (the inset shows the linear relationship between the (F_0_ − F)/F_0_ and Cr(VI) concentration within the range of 0.1–60 μM) and Fe^3+^ concentration (**d**) (the inset shows the linear relationship between the (F_0_ − F)/F_0_ and Fe^3+^ concentration within the range 0.05–100 μM), respectively. (The error bar was not obvious due to the small fluctuation of data).

The sensing sensitivity of FW-CDs towards Cr(VI) and Fe^3+^ was further investigated by adding various concentrations of these two ions. As depicted in Figure 4a, the fluorescence intensity of FW-CDs continuously decreased with the increase in Cr(VI) concentration in the range of 0–500 µM, and the fluorescent quenching ratio ((F_0_ − F)/F_0_) showed a good linearity with the Cr(VI) concentrations from 0.1 to 60 µM (Figure 4b). The corresponding LOD was calculated as 0.07 µM through the formula 3σ/slope, which was much lower than the allowable maximum limit of Cr(VI) concentration (9.6 µM) in Cr(VI)-containing industrial effluent in China. As for the determination of Fe^3+^, a good linearity (Figure 4d) between the fluorescent quenching ratio and Fe^3+^ concentrations was also obtained in the range of 0.05–100 µM with an LOD of 0.15 µM, which was far below the allowable value (5.4 µM). To our knowledge, among all the reported CD-based Cr(VI)/Fe^3+^ dual-mode fluorescent sensors, the LODs of the FW-CD-based fluorescence probe are the lowest recorded values (Table 1). Furthermore, Table 2 shows that the prepared FW-CD-based nanoprobe also has higher sensitivity for detecting Cr(VI) and Fe^3+^ when compared with the recently reported different types of fluorescence CDs for detecting Cr(VI) and Fe^3+^. Taken together, the above results verified that the constructed FW-CD-based nanoprobe not only could be used for the parallel and independent determination of Cr(VI) and Fe^3+^ with high specificity, but also possesses excellent dual sensing performance with ultralow LODs.

### 3.4. Possible Quenching Mechanism of Cr(VI) and Fe^3+^ to FW-CDs

To clarify the precise quenching mechanisms of Cr(VI) and Fe^3+^ to FW-CDs, the fluorescence lifetimes of FW-CDs before and after adding Cr(VI) and Fe^3+^ were measured, respectively. As shown in Supplementary Figure S4, the average fluorescence lifetime (τ_0_) of FW-CDs barely changed in the presence and absence of Cr(VI) and Fe^3+^, and was calculated to be around 6.6 ns, thus excluding the possibilities of dynamic quenching mechanisms, i.e., the photoinduced electron transfer (PET) and Forster resonant energy transfer (FRET) processes. Subsequently, to further clarify the quenching mechanism, the UV-vis absorption spectra were investigated.

**Figure 5:**
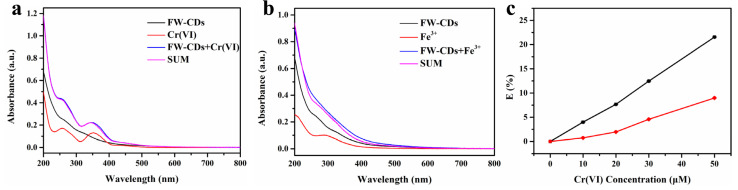
(**a**) UV–vis absorption spectra of FW-CDs, Cr(VI) and the FW-CDs/Cr(VI) system, and the summed absorption spectrum of FW-CDs and Cr(VI), respectively. (**b**) UV–vis absorption spectra of FW-CDs, Fe^3+^ and the FW-CDs/Fe^3+^ system, and the summed absorption spectrum of FW-CDs and Fe^3+^, respectively. (**c**) Suppressed efficiency (E, %) of observed (black line, E_obsd_) and corrected (red line, E_cor_) fluorescence intensity, λ_ex_ = 375 nm, λ_em_ = 445 nm. (The error bar was not obvious due to the small fluctuation of data).

As exhibited in Figure 5a, the summed absorption spectrum of FW-CDs and Cr(VI) was highly consistent with the UV-vis absorption band of FW-CDs/Cr(VI) mixed solution, demonstrating that no new substance was formed during the quenching process and the possibility of SQE was ruled out. Because of the fact that the excitation wavelength of FW-CDs had a large overlap with the absorption spectrum of Cr(VI), the quenching mechanism of FW-CDs caused by Cr(VI) was considered to be an inner filter effect (IFE). To further confirm the deduction, the corrected fluorescence intensity (F_cor_) of FW-CDs before considering IFE was calculated based on the Parker equation, the data processing of which was conducted following the same procedures and using the same parameters as our previous work [3]. As depicted in Figure 5c, when the content of Cr(VI) increased, both the correction factor (CF = F_cor_/F_obsd_) values and the discrepancy between E_obsd_ and E_cor_ continuously increased, which indicated that IFE takes up a very large proportion of the observed total suppression efficiency in the fluorescence-quenching process. Therefore, IFE plays a dominant role in the quenching process of Cr(VI) to FW-CDs. Table 3 lists the parameters utilized to calculate IFE of Cr(VI) on the fluorescence of FW-CDs.

Similarly, it is shown in Figure 5b that the summed absorption spectrum of the FW-CDs and Fe^3+^ and the UV-vis absorption band of FW-CDs/Fe^3+^ mixed solution were mismatched, which suggests the formation of the ground-state complex between Fe^3+^ and the FW-CDs. It is also important to note that the excitation wavelength of FW-CDs scarcely overlapped with the absorption band of Fe^3+^, indicating that the IFE was not the primary cause in the fluorescence-quenching process of Fe^3+^ to the FW-CDs. Furthermore, FTIR was utilized to further prove the formation of the ground-state complex between Fe^3+^ and the functional groups of FW-CDs. As shown in Supplementary Figure S5, the difference of FTIR spectra between the FW-CDs and the FW-CDs/Fe^3+^ mixture, and especially the variation of absorption band intensities around 3415 cm^−1^ and 1400 cm^−1^, which were related to -OH, -NH and -COOH, further demonstrated the formation of the ground-state complex between the hydrophilic functional groups of the FW-CDs and Fe^3+^. Specifically, the outstanding selectivity of the FW-CDs to Fe^3+^ could be attributed to the faster chelating kinetics and the strong binding preference of Fe^3+^ towards the hydroxyl groups and carboxyl groups on the surface of the FW-CDs compared with other metal ions. These factors could lead to the nonradiative electron transfer from the excited state of the FW-CDs, causing a fluorescence quenching of the FW-CDs [68]. Collectively, SQE was the principal quenching mechanism of Fe^3+^ to the FW-CDs.

### 3.5. Quantitative and Parallel Determination of Cr(VI) and Fe^3+^ in Environmental Water Samples

To evaluate the feasibility of the FW-CDs nanoprobe, it was utilized for the quantitative and simultaneous detection of Cr(VI) and Fe^3+^ in real environmental water specimens (tap, lake and river water). A quantity of 5 µM Fe^3+^ and various amounts of Cr(VI) (5, 10 and 20 µM) were severally spiked into environmental water specimens. Subsequently, the FW-CD-based independent detection of Cr(VI) was conducted using the NaF masking strategy. As listed in Table 4, both the FW-CD nanoprobe and ICP-OES found no Cr(VI) in the unspiked water specimens. The spiked recoveries of Cr(VI) obtained by the FW-CDs nanoprobe were in a range of 92.6% to 116.2% and the relative standard deviations (RSD, *n* = 3) were lower than 1.8%, which was close to the ICP-OES results. Furthermore, 10 µM Cr(VI) and different amounts of Fe^3+^ (5, 10 and 20 µM) were severally spiked into the environmental water specimens. Similarly, the BaCl_2_ masking method was utilized for the FW-CD-based independent detection of Fe^3+^ ions. Table 5 shows that both the FW-CDs nanoprobe and ICP-OES detected trace quantities of Fe^3+^ in the unspiked specimens with values that were close to each other. The spiked recoveries of Fe^3+^ obtained by the FW-CD nanoprobe were in the range of 84.8% to 104.8% with RSDs lower than 8.0%, which were consistent with those of ICP-OES. Therefore, the FW-CDs nanoprobe exhibited good accuracy and high availability for quantitatively and parallelly determining Cr(VI) and Fe^3+^ in real water specimens. Furthermore, the good stability of the FW-CDs in three kinds of real water specimen is illustrated in Supplementary Figure S6.

### 3.6. Quantitative Determination of Cr(VI) in Industrial Effluent

The developed FW-CDs nanoprobe was further adopted to quantitatively detect Cr(VI) in industrial effluent, and the standard curve of Cr(VI) obtained by the above sensing platform was utilized to calculate a Cr(VI) concentration that is unknown in the industrial effluent. The Cr(VI) content obtained by FW-CDs nanoprobe was calculated to be about 31.0 µM, corresponding to the measurement result of ICP-OES (30.4 µM). Thus, the developed FW-CDs nanoprobe is highly available and reliable for the quantitative determination of Cr(VI) in industrial effluent.

### 3.7. Quantitative Determination of Fe^2+^ in Oral Iron Supplement

The content of Fe^2+^ in commercial oral ferrous sulfate tablets was also determined by the developed FW-CDs nanoprobe. After the Fe^2+^ ions contained in the samples were completely oxidized to Fe^3+^ by hydrogen peroxide and diluted 100 times with hydrochloric solution, the Fe^3+^ concentration in the diluent was determined to be about 10.8 µM using the FW-CD nanoprobe (the negligible interference of hydrogen peroxide with the FW-CDs is shown in Supplementary Figure S7). For comparison, the nominal amount of Fe^3+^ was equivalent to 11.6 µM and the quantitative result determined by ICP-OES was about 10.2 µM, which is in line with the result obtained by the FW-CD-based sensing system. Therefore, the FW-CDs nanoprobe also has a huge application potential for the quantitative determination of Fe^3+^ in iron-containing drugs.

### 3.8. On-Site Semi-Quantitative Visual Determination of Cr(VI) and Fe^3+^ in Real Samples

Encouraged by the superior sensitivity, specificity and availability of FW-CDs for quantitatively and parallelly sensing Cr(VI) and Fe^3+^, a more convenient, high efficiency and inexpensive method for the on-site semi-quantitative visual determination of Cr(VI) and Fe^3+^ was developed on the basis of an FW-CD-based fluorescent hydrogel sensor consisting of agarose and FW-CDs. The freshly fabricated FW-CD-doped hydrogels emitted uniformly blue fluorescence when illuminated with a UV lamp and no leakage of FW-CDs was found when the hydrogels were immersed in ultrapure water, indicating the uniform and firm immobilization of FW-CDs in the hydrogel matrix. The influence of the content of FW-CDs doped in the hydrogels on the visual sensing performance was investigated. The hybrid hydrogels containing various amounts of FW-CDs (0.5, 1 and 2 mg/mL) were immersed in the test solutions containing different concentrations of Cr(VI) or Fe^3+^. As shown in Supplementary Figure S8, the hydrogel containing 1 mg/mL FW-CDs had the optimum visual performance for preferably distinguishing the Cr(VI) and Fe^3+^ contents with the naked eye. Therefore, the hydrogel containing 1 mg/mL FW-CDs was chosen for the following experiments. Additionally, the effect of incubation time on the fluorescence change of the hydrogels was also studied. As illustrated in Supplementary Figure S9, the fluorescence intensity of the hydrogels hardly decreased after being incubated with 100 µM Cr(VI) or Fe^3+^ ions for 30 min. Therefore, 30 min was selected as the incubation time for subsequent operations.

**Figure 6:**
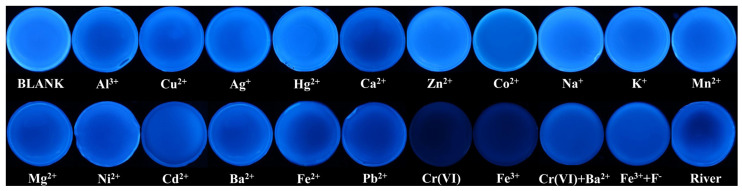
The selectivity and anti-interference capability of the FW-CD-based hydrogel sensor. The concentration of each metal ion was 100 μM.

**Figure 7:**
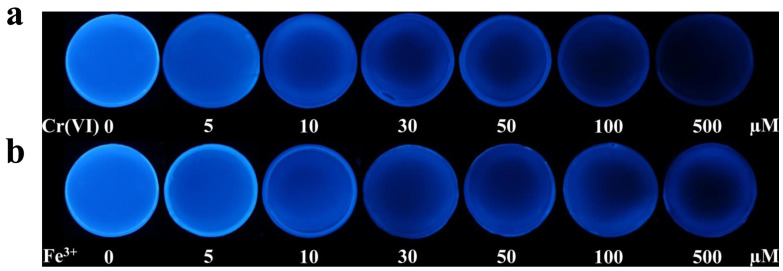
The fluorescence-quenching effects of FW-CD hydrogels in the presence of different concentrations of Cr(VI) (**a**) and Fe^3+^ (**b**) at 30 min.

In order to verify the feasibility of this visual sensing system, the FW-CD-doped hydrogels were immersed in the test solutions containing different kinds of metal ions (100 µM). As shown in Figure 6, after soaking for 10 min, the fluorescence of FW-CD-based hydrogels was distinctly weakened in the solution of Cr(VI) and Fe^3+^, while only a slight quenching was observed in the presence of other kinds of metal ions. Thus, the FW-CD-based hydrogel sensor possesses excellent selectivity for the determination of Cr(VI) and Fe^3+^. The sensitivity of the FW-CD-based hydrogel sensor was evaluated as well. Briefly, the FW-CD-doped hydrogels were immersed in the test solutions containing various amounts of Cr(VI) or Fe^3+^. As shown in Figure 7, with the increase in Cr(VI) or Fe^3+^ content from 5 to 500 µM, their fluorescence intensity continuously decreased. The fluorescence changes of the hydrogels caused by 10 µM Cr(VI) or 5 µM Fe^3+^ in particular, which were close to their maximum limit, could be easily distinguished by the naked eye, implying good sensitivity of the FW-CD-based hydrogel sensor. Next, the environmental water sample was utilized to assess the anti-interference capability of the visual sensing system. As exhibited in Figure 6, a slight change was observed when the FW-CD-based hydrogels were placed in the river water sample, indicating that the potential interference in a real complex sample caused by other metal ions on the visual sensing could be ignored. Furthermore, the effectiveness of the dual-masking strategy for avoiding the mutual interference of Cr(VI) and Fe^3+^ in the visual detection process was further explored. As exhibited in Figure 6, the quenched fluorescence of FW-CD-based hydrogels induced by Cr(VI) and Fe^3+^ was well recovered after adding Ba^2+^ and F^−^, respectively, suggesting that the FW-CD-based fluorescent hydrogel sensor holds huge application value for the parallel and visual sensing of Cr(VI) and Fe^3+^.

The semi-quantitative visual determination of Cr(VI) and Fe^3+^ in real samples was conducted ulteriorly. As depicted in Supplementary Figure S10, the fluorescence intensity of FW-CD-based hydrogels soaked in the Cr(VI)-containing industrial effluent was apparently weaker than those incubated with 10 µM Cr(VI) reference solution, suggesting an excess of Cr(VI) content in the sample. Additionally, the fluorescence of FW-CD-based hydrogels soaked into the iron supplement diluent was close to that of the reference solution containing 10 µM Fe^3+^, indicating that the content of Fe^3+^ in the sample was basically consistent with the nominal content of Fe^3+^ (11.6 µM). Thus, the as-prepared FW-CD-based hydrogel sensor possesses potential for the on-site semi-quantitative visual determination of Cr(VI) and Fe^3+^ in complex specimens.

## 4. Conclusions

In summary, a novel multifunctional fluorescent sensor based on waste-biomass-derived FW-CDs has been rationally prepared by co-using the dual-functional integration and dual-masking strategies for the parallel visual detection of Cr(VI) and Fe^3+^ with high specificity and sensitivity. Profiting from the diverse surface functional groups of the fabricated biomass-derived FW-CDs, the synthesized FW-CDs had an excellent parallel sensing ability toward Cr(VI) and Fe^3+^, which was much lower than the corresponding values of all the previously reported CD-based Cr(VI)/Fe^3+^ dual-mode sensors. By co-utilizing BaCl_2_ and NaF as the masking agents, the latent mutual interference between Cr(VI) and Fe^3+^ could be effectively eliminated to achieve the independent analysis of Cr(VI) and Fe^3+^ with high specificity. Meanwhile, the obtained FW-CDs were successfully applied for the quantitative detection of Cr(VI) and Fe^3+^ in environmental water samples, industrial effluent and iron supplement with high precision and satisfactory recovery. Moreover, a novel FW-CD-based fluorescent hydrogel sensor was fabricated for the first time for the parallel and semi-quantitative visual detection of Cr(VI) and Fe^3+^ in real samples. This study not only provides a reliable path to promote the evolution of CD-based multifunctional fluorescent sensors from tandem sensing with strong interdependence to parallel detection without mutual interference, but also holds huge application potential for visual sensing, accurate diagnosis and environmental monitoring.

## Figures and Tables

**Table 1:** Comparison of the sensing performances of different CD-based Cr(VI)/Fe^3+^ dual-mode fluorescent sensors.

Samples	Precursors of CDs	Linear Range (µM)	LOD (µM)	Ref.
Cr(VI)/Fe^3+^	Waste tea	1.9–115.4/53.5–267.9	0.81/0.15	[43]
	Natural microcline	0–68/0–68	4/19	[44]
	Citric acid/acrylamide	8–160/1–40, 2–180	2.10/0.87	[45]
	DECDs	10–100/10–100	3.2/4.1	[61]
	Flowers of wintersweet	0.1–60/0.05–100	0.07/0.15	This work

**Table 2:** Comparison of different CD-based fluorescent probes for Cr(VI) and Fe^3+^ detection.

Samples	Precursors of CDs	Linear Range (µM)	LOD (µM)	Ref.
Cr(VI)	Enokitake mushroom	10–50	0.73	[58]
	Citric acid/ethylenediamine	0–10	0.26	[62]
	Pomelo	1–40	0.52	[63]
	Citric acid and glycine	5–200	4.16	[64]
	af-CDs	0.8–80	0.24	[65]
	Flowers of wintersweet	0.1–60	0.07	This work
Fe^3+^	pf-CDs	0.8–80	0.16	[65]
	Sweet potato	1–100	0.32	[66]
	Seville orange	33–133	0.53	[67]
	Dwarf banana peel	5–25	0.66	[23]
	Fish scales	1–78	0.54	[42]
	Flowers of wintersweet	0.05–100	0.15	This work

**Table 3:** Parameters utilized to calculate IFE of Cr(VI) on the fluorescence of FW-CDs.

Cr(VI) (μM)	A_ex_	A_em_	CF	F_obsd_	F_cor_	E_obsd_	E_cor_	F_cor_/F_cor,0_
0	0.031	0.015	1.050	905.48	950.67	0	0	1
10	0.055	0.021	1.086	869.19	943.63	0.040	0.003	0.992
20	0.075	0.025	1.115	835.93	931.97	0.077	0.020	0.980
30	0.094	0.030	1.145	792.54	907.18	0.125	0.046	0.954
50	0.143	0.038	1.218	710.34	865.22	0.216	0.090	0.910

**Table 4:** Recoveries of Cr(VI) in environmental water samples using the FW-CDs nanoprobe and ICP-OES, respectively.

Sample	Added (µM)	Found (µM)	Found (µM) (ICP-OES)	Recovery (%)	Recovery (%) (ICP-OES)
Tap	0	ND *	ND	-	-
	5	5.81 ± 1.1 *	4.96 ± 2.6	116.2 ± 1.1	99.2 ± 2.6
	10	9.45 ± 0.3	10.06 ± 1.7	94.5 ± 0.3	100.6 ± 1.7
	20	20.07 ± 0.3	19.98 ± 0.4	100.4 ± 0.3	99.9 ± 0.4
Lake	0	ND	ND	-	-
	5	4.63 ± 1.8	5.02 ± 2.9	92.6 ± 1.8	100.4 ± 2.9
	10	10.24 ± 0.3	9.76 ± 1.1	102.4 ± 0.3	97.6 ± 1.1
	20	19.97 ± 0.6	19.32 ± 0.9	99.9 ± 0.6	96.6 ± 0.9
River	0	ND	ND	-	-
	5	5.12 ± 1.6	4.50 ± 1.1	102.4 ± 1.6	90.0 ± 1.1
	10	10.11 ± 0.7	9.68 ± 1.1	101.1 ± 0.7	96.8 ± 1.1
	20	19.91 ± 0.5	19.12 ± 0.9	99.6 ± 0.5	95.6 ± 0.9

* Each sample was measured three times in parallel; ND: not detected.

**Table 5:** Recoveries of Fe^3+^ in environmental water samples using the FW-CDs nanoprobe and ICP-OES, respectively.

Sample	Added (µM)	Found (µM)	Found (µM) (ICP-OES)	Recovery (%)	Recovery (%) (ICP-OES)
Tap	0	0.15 ± 8.0 *	0.10 ± 6.0	-	-
	5	5.12 ± 1.2	5.20 ± 1.9	99.4 ± 1.2	102.0 ± 1.9
	10	10.12 ± 5.7	10.66 ± 3.2	99.7 ± 5.7	105.6 ± 3.2
	20	19.91 ± 1.4	20.43 ± 0.6	98.8 ± 1.4	101.7 ± 0.6
Lake	0	0.18 ± 2.1	0.06 ± 8.4	-	-
	5	4.42 ± 6.0	4.41 ± 4.7	84.8 ± 6.0	87.0 ± 4.7
	10	10.30 ± 2.8	10.34 ± 1.9	101.2 ± 2.8	102.8 ± 1.9
	20	19.91 ± 1.6	20.28 ± 2.5	98.7 ± 1.6	101.1 ± 2.5
River	0	0.08 ± 2.6	0.03 ± 3.9	-	-
	5	5.32 ± 1.2	4.92 ± 1.1	104.8 ± 1.2	97.8 ± 1.1
	10	9.68 ± 3.0	9.85 ± 5.2	96.0 ± 3.0	98.2 ± 5.2
	20	20.08 ± 0.9	19.70 ± 1.0	100.0 ± 0.9	98.4 ± 1.0

* Each sample was measured three times in parallel.

## Data Availability

The data presented in this study are available on request from the corresponding authors.

## Supplementary Materials

Figure S1. XRD patterns of the prepared FW-CDs. Figure S2. (**a**) Full XPS spectrum of FW-CDs. (**b**) High-resolution C 1s XPS spectrum. (**c**) O 1s XPS spectrum. (**d**) N1s XPS spectrum of FW-CDs. Figure S3. (**a**) The fluorescence intensity of FW-CDs at different pH values. (**b**) Effect of NaCl concentration on the fluorescence intensity of FW-CDs. (**c**) Photostability of FW-CDs irradiated by xenon lamp for different amounts of time. (The error bar was not obvious due to the small fluctuation of data.) Figure S4. Fluorescence decay curve of as-prepared FW-CDs in the absence and presence of Cr(VI) (**a**) and Fe^3+^ (**b**) with excitation at 375 nm. Figure S5. FTIR spectra of the FW-CDs and FW-CDs/Fe^3+^ mixture. Figure S6. Photostability of FW-CDs irradiated by xenon lamp for different amounts of time in tap (**a**), lake (**b**), river water (**c**). (The error bar was not obvious due to the small fluctuation of data.) Figure S7. The fluorescence intensity changes of FW-CDs in the absence and presence of 500 μM H_2_O_2_. Figure S8. Comparison of the fluorescence intensity of FW-CDs hydrogels containing different concentrations of FW-CDs in the presence of different amounts of Cr(VI) (**a**) and Fe^3+^ (**b**). Figure S9. The fluorescence-quenching effect of FW-CD hydrogels in 100 μM Cr(VI) (**a**) and Fe^3+^ (**b**) solution at different incubation times. Figure S10. (**a**) Fluorescence comparison between FW-CDs hydrogels, FW-CDs hydrogels incubated with 10 μM Cr(VI) and FW-CDs hydrogels incubated with wastewater, respectively. (**b**) Fluorescence comparison between FW-CDs hydrogels, FW-CDs hydrogels incubated with 10 μM Fe^3+^ and FW-CDs hydrogels incubated with iron supplement, respectively.

## Sample Availability

Samples of the compounds are available from the authors.
