# Supplementary material for: Sustainable and Green Synthesis of Waste-Biomass-Derived Carbon Dots for Parallel and Semi-Quantitative Visual Detection of Cr(VI) and Fe3+
Source: Molecules. 2022 Feb 13;27(4):1258. doi: 10.3390/molecules27041258 (PMC8876948; doi:10.3390/molecules27041258)
Supplement: Supplementary file 1 [file molecules-27-01258-s001.zip › molecules-1579749-supplementary.pdf]

*Supplementary Material*

# Sustainable and Green Synthesis of Waste-Biomass-Derived Carbon Dots for Parallel and Semi-Quantitative Visual Detection of Cr(VI) and $Fe^{3+}$

**Lan Xia [1,†], Xiuju Li [2,†], Yuanhua Zhang [1], Kai Zhou [3], Long Yuan [1], Rui Shi [4,*], Kailian Zhang [1] and Qifeng Fu [1,*]**

[1] Department of Pharmaceutical Analysis, School of Pharmacy, Southwest Medical University, Luzhou 646000, China; 20200599120020@stu.swmu.edu.cn (L.X.); 20190599120011@stu.swmu.edu.cn (Y.Z.); 20210599120022@stu.swmu.edu.cn (L.Y.); zkl66@swmu.edu.cn (K.Z.)

[2] School of Pharmacy, Tongren Polytechnic College, Tongren 554300, China; lxj_trzy@163.com

[3] Analytical and Testing Center, Chongqing University, Chongqing, 401331, China; zhoukai2016@cqu.edu.cn

[4] Emergency Department, The Affiliated Traditional Chinese Medicine Hospital of Southwest Medical University, Southwest Medical University, Luzhou, Sichuan 646000, China

* Correspondence: Sherry0503s@163.com (R.S.); fuqifeng@swmu.edu.cn (Q.F.); Tel.: +86-830-25-23-333 (R.S.); Tel.: +86-830-31-61-291 (Q.F.)

† These authors contributed equally to this work.

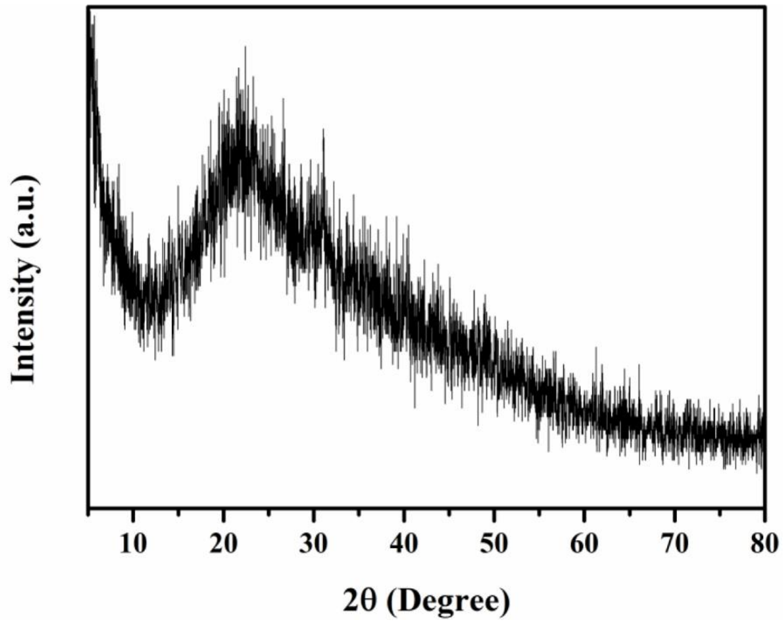


**Figure S1.** XRD patterns of the prepared FW-CDs.

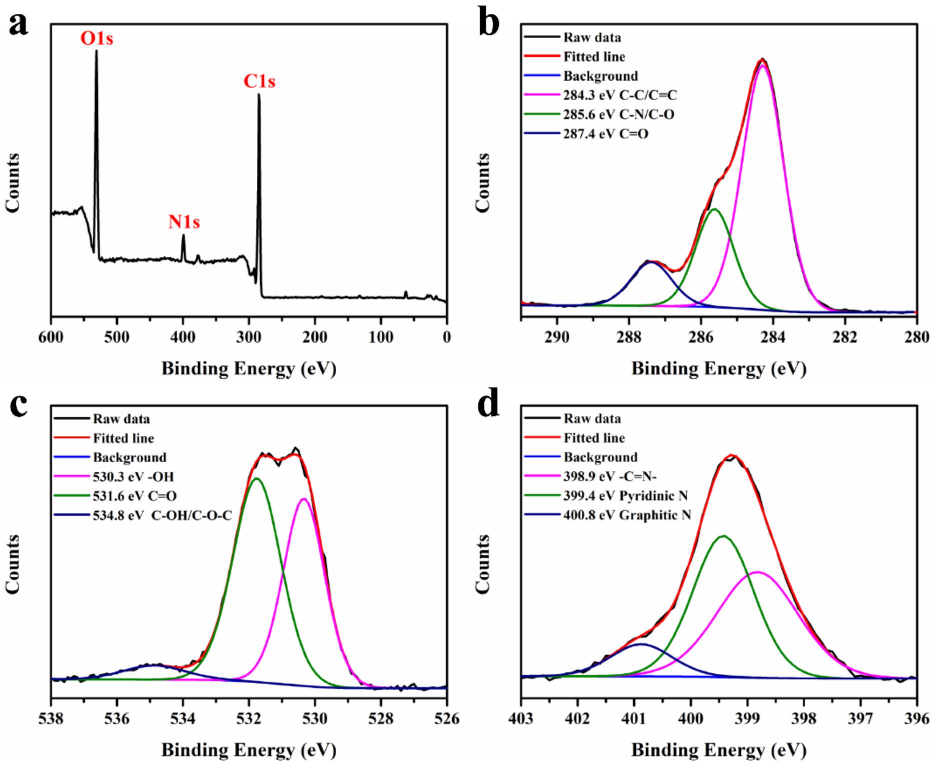


**Figure S2.** (**a**) Full XPS spectrum of FW-CDs; (**b**) High-resolution C 1s XPS spectrum; (**c**) O 1s XPS spectrum; (**d**) N1s XPS spectrum of FW-CDs.

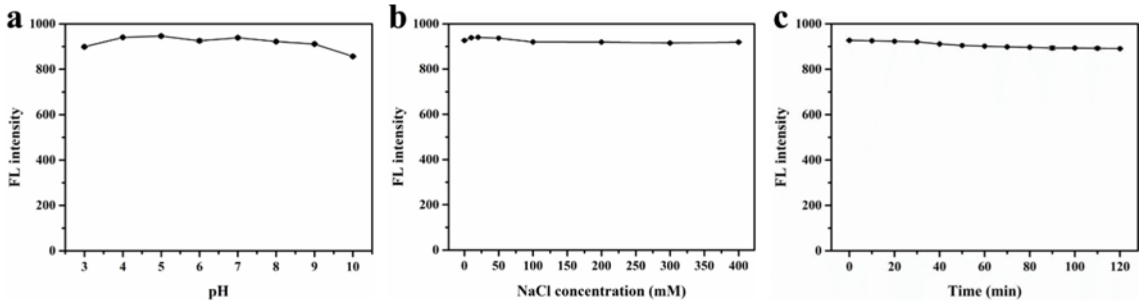


**Figure S3.** (**a**) The fluorescence intensity of FW-CDs at different pH values; (**b**) Effect of NaCl concentration on the fluorescence intensity of FW-CDs; (**c**) Photostability of FW-CDs irradiated by xenon lamp for different amounts of time. (The error bar was not obvious due to the small fluctuation of data).

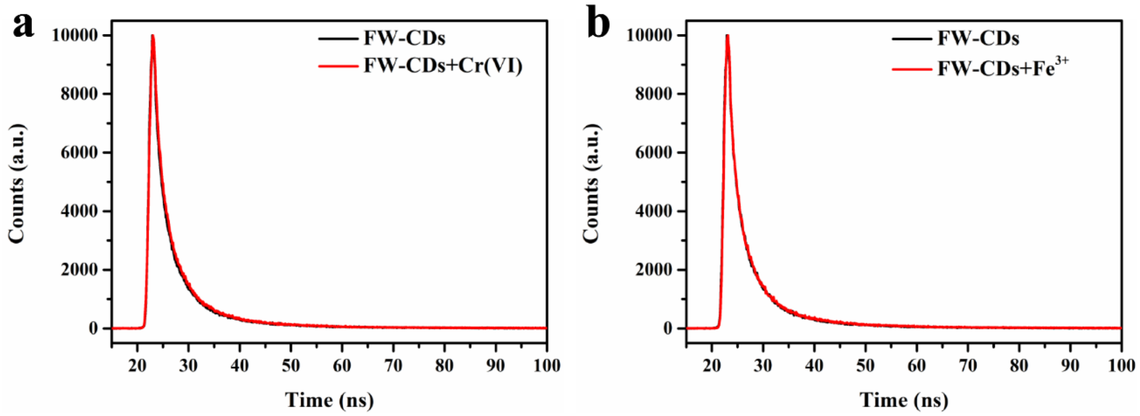


**Figure S4.** Fluorescence decay curve of as-prepared FW-CDs in the absence and presence of Cr(VI) (**a**) and $Fe^{3+}$ (**b**) with excitation at 375 nm.

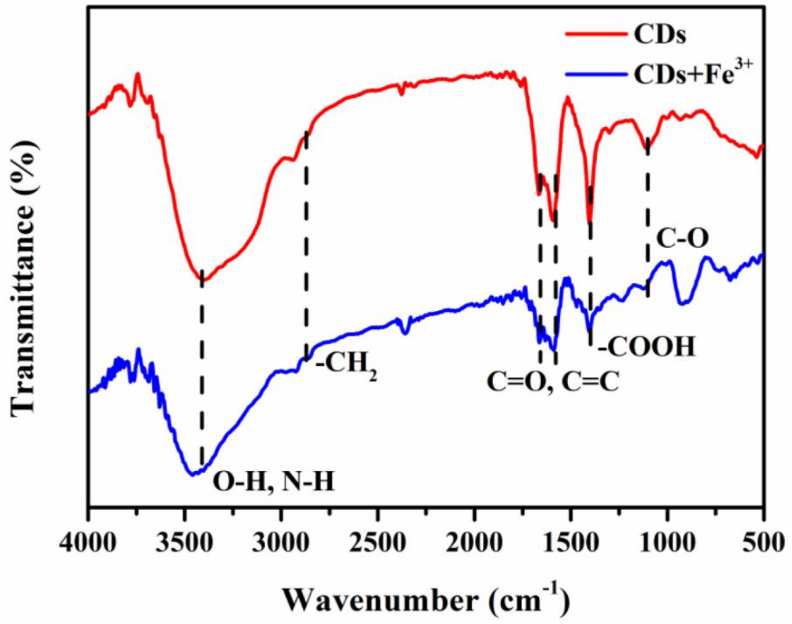


**Figure S5.** FTIR spectra of the FW-CDs and FW-CDs/$Fe^{3+}$ mixture.

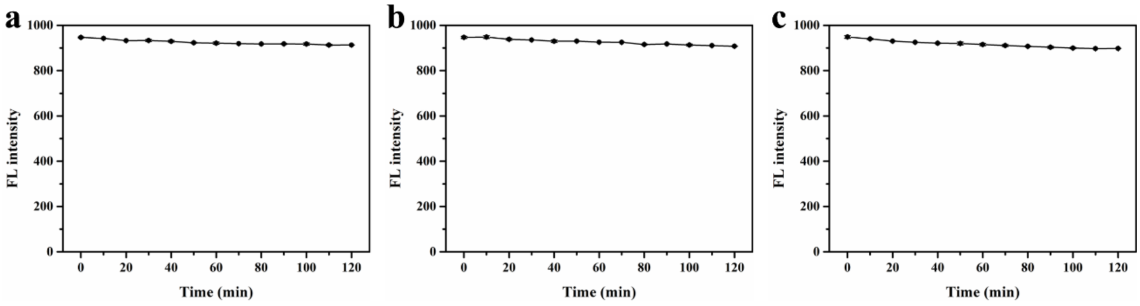


**Figure S6.** Photostability of FW-CDs irradiated by xenon lamp for different amounts of time in tap (**a**), lake (**b**), river water (**c**). (The error bar was not obvious due to the small fluctuation of data).

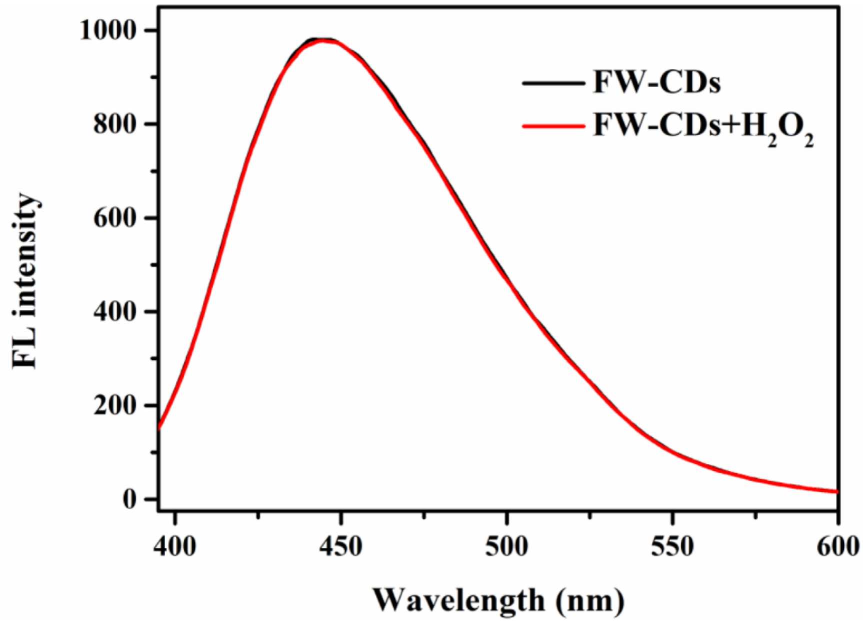


**Figure S7.** The fluorescence intensity changes of FW-CDs in the absence and presence of 500 μM $H_2O_2$.

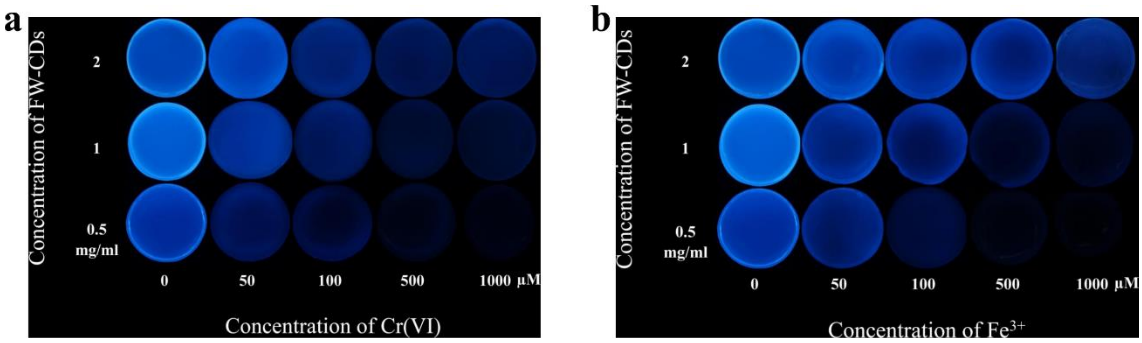


**Figure S8.** Comparison of the fluorescence intensity of FW-CDs hydrogels containing different concentrations of FW-CDs in the presence of different amounts of Cr(VI) (a) and $Fe^{3+}$ (b).

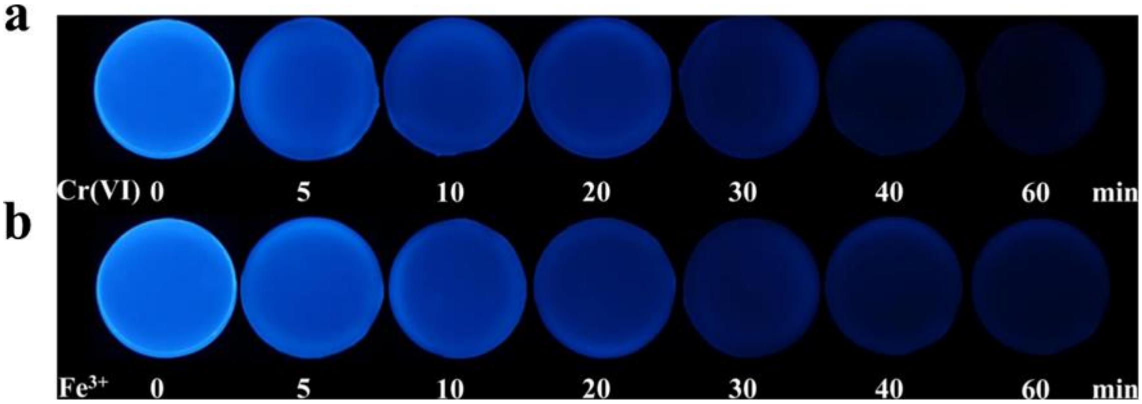


**Figure S9.** The fluorescence quenching effect of FW-CD hydrogels in 100 μM Cr(VI) (**a**) and $Fe^{3+}$ (**b**) solution at different incubating time.

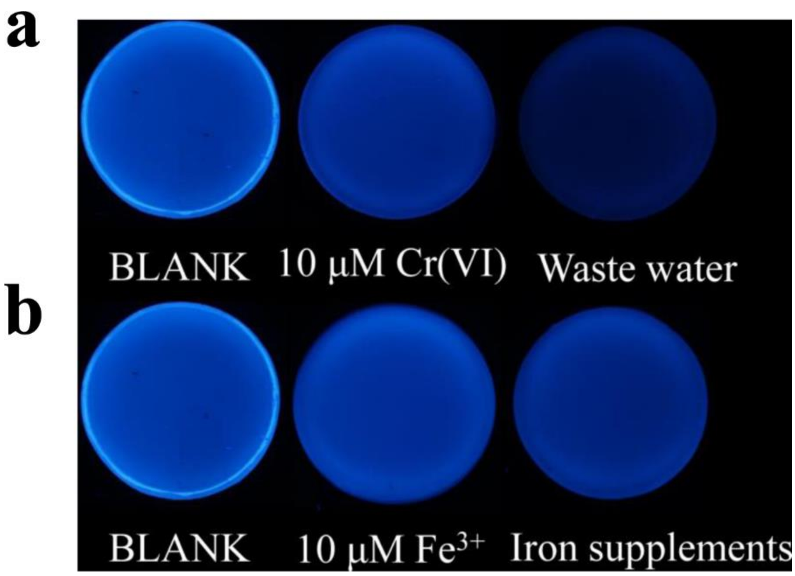


**Figure S10.** (**a**) Fluorescence comparison between FW-CDs hydrogels, FW-CDs hydrogels incubated with 10 μM Cr(VI), and FW-CDs hydrogels incubated with wastewater, respectively; (**b**) Fluorescence comparison between FW-CDs hydrogels, FW-CDs hydrogels incubated with 10 μM $Fe^{3+}$, and FW-CDs hydrogels incubated with iron supplement, respectively.
